# Gastroesophageal Reflux in patients with Recurrent Laryngeal Papillomatosis

**DOI:** 10.1016/S1808-8694(15)31068-5

**Published:** 2015-10-22

**Authors:** Shirley Shizue Nagata Pignatari, Raquel Ysabel Guzmán Liriano, Melissa A.G. Avelino, José Ricardo Gurgel Testa, Reginaldo Fujita, Eduardo Kutchell De Marco

**Affiliations:** 1 Adjunct Professor, master's degree and doctorate at the Otorhinolaryngology and Head & Neck Surgery Department, Discipline of Pediatric Otorhinolaryngology, Sao Paulo Federal University - Paulista Medical School, Adjunct Professor - Head of the Pediatric Otorhinolaryngological Discipline.; 2 Master's degree on Science at the Otorhinolaryngology and Head & Neck Surgery Department, Discipline of Pediatric Otorhinolaryngology, Sao Paulo Federal University - Paulista Medical School Otorhinolaryngologist.; 3 Master's degree and doctorate on Science at the Otorhinolaryngology and Head & Neck Surgery Department, Discipline of Pediatric Otorhinolaryngology, Sao Paulo Federal University - Paulista Medical School, Otorhinolaryngologist.; 4 Adjunct Professor, Master's degree and doctor at the Otorhinolaryngology and Head & Neck Surgery Department, Discipline of Pediatric Otorhinolaryngology, Sao Paulo Federal University - Paulista Medical School.; 5 Adjunct Professor, Master's degree and doctor at the Otorhinolaryngology and Head & Neck Surgery Department, Discipline of Pediatric Otorhinolaryngology, Sao Paulo Federal University - Paulista Medical School.; 6 Medical doctor, gastroenterologist, Director of the Physiology and Motility Unit - pHmetry and manometry unit at the Real e Benemerita Sociedade Portuguesa de Beneficencia de Sao Paulo - São Joaquim Hospital.; 7 Otorhinolaryngology and Head & Neck Surgery Department, Discipline of Pediatric Otorhinolaryngology, Sao Paulo Federal University - Paulista Medical School.

**Keywords:** children, laryngeal papillomatosis, gastroesophageal reflux

## Summary

**E**vidence of a relation between gastroesophaeal reflux and pediatric respiratory disorders increases every year. Many respiratory symptoms and clinical conditions such as stridor, chronic cough, and recurrent pneumonia and bronchitis appear to be related to gastroesophageal reflux. Some studies have also suggested that gastroesophageal reflux may be associated with recurrent laryngeal papillomatosis, contributing to its recurrence and severity. **Aim:** the aim of this study was to verify the frequency and intensity of gastroesophageal reflux in children with recurrent laryngeal papillomatosis. **Material and Methods:** ten children of both genders, aged between 3 and 12 years, presenting laryngeal papillomatosis, were included in this study. The children underwent 24-hour double-probe pH-metry. **Results:** fifty percent of the patients had evidence of gastroesophageal reflux at the distal sphincter; 90% presented reflux at the proximal sphincter. **Conclusion:** the frequency of proximal gastroesophageal reflux is significantly increased in patients with recurrent laryngeal papillomatosis.

## INTRODUCTION

Recurrent laryngeal papillomatosis (RLP) is characterized by the presence of usually recurrent benign laryngeal epithelial verrucous lesions. Morbidity is high because these confluent lesions cause progressive dysphonia (voice disorders) and dyspnea (respiratory changes). These lesions may lead to respiratory failure and even death due to obstruction. Recurrence has frustrated otorhinolaryngologists; although benign, this disease is one of the most difficult to control in the specialty.^1^ It is caused by the HPV virus - usually types 6 and 11 - affecting both adults and children.^2^

HPV infection is transmitted by intimate contact and is facilitated by mild trauma at the inoculation site. It may result from direct contact with another person or, less frequently, by self-inoculation. It appears that viral transmission of laryngeal papillomatosis takes place in the birth canal or through sexual contact.^2^,^3^ A few papers have demonstrated that cesarean sections do not prevent laryngeal papillomatosis or fetal contamination, which may occur by transmission through blood or amniotic fluid.^4^

Estimates suggest that there are around 1,500 to 2,500 new cases of RLP each year in the USA.^5^ Recurrences and specific therapy have been studied for decades, and are still a challenge.

Gastroesophageal reflux is a physiological phenomenon in neonates and children below 18 months. Depending on the frequency and duration, reflux may result in chronic airway inflammation, giving rise to a clinical condition which receives the name gastroesophageal reflux disease (GERD). According to the World Gastroenterology Conference, the term gastroesophageal reflux disease should be used in persons that are at risk for physical complications of gastroesophageal reflux, or that clinically present decreased health and a worsened quality of life due to reflux-related symptoms after adequate recognition of the benign nature of these symptoms.^6^

There is growing evidence each year that gastroesophageal reflux contributes to airway disorders in children, and may lead to respiratory symptoms such as stridor, chronic coughing, repeat pneumonia, and chronic bronchitis.^7^

Borkowski et al. in 1999 first suggested a correlation between RLP and gastroesophageal reflux by showing that controlling reflux resulted in reduced growth of laryngeal papillomas.^8^ At the same time other authors started to note that gastroesophageal reflux was very frequent in RLP patients, and that this condition could also be associated with other laryngeal disorders such as recurrent croup, subglottal stenosis and vocal nodules.^9^,^10^

The HPV virus in RLP causes recurrence, and may remain latent in the laryngeal mucosa for an unknown time. Factors responsible for HPV virus activation are not yet clear. Extraesophageal reflux has been considered one of the factors responsible for the difficulty in controlling laryngeal papilloma recurrence.^11^

Our aim was to investigate the association and intensity of gastroesophageal reflux in children with RLP using double probe 24-hour esophageal pH monitoring.

## MATERIAL AND METHOD

We selected ten children of both genders, aged between 3 and 12 years (5 male and 5 female), with a diagnosis of laryngeal papillomatosis.

Patients were recruited from the Laryngeal Papillomatosis Outpatient Unit of the pediatric otorhinolaryngology department at the UNIFESP-EPM.

Persons legally responsible for the patients read and signed an informed consent form that had previously been approved by the Research Ethics Committee of the Sao Paulo Hospital - UNIFESP. After signing the free informed consent form, patients underwent double probe esophageal pH monitoring at the Beneficencia Portuguesa Hospital.

### Esophageal pH monitoring test:

Patients were asked not to take any H2 blocking agent or proton pump inhibitor during seven days prior to esophageal pH monitoring. Monitoring lasted at least 20 hours. Children remained in the hospital; they received their usual diet, and were able to exercise normally. Their mothers annotated meals and rest periods. A nutritionist adapted the type of food for each age group, and only tea, soft drinks and seasoning were restricted.

Gastroesophageal reflux was measured in all patients by continuous monitoring. The pH electrode was introduced nasally, after anesthesia with lidocaine gel 2%. The electrode probe gauge was 2.1mm; there were two antimony sensors and an external reference sensor. The probe was semi-disposable and was cleaned externally with enzyme soap (Endozyme®) and disinfected with a glutaraldehyde 2% solution for 15 minutes. The antimony electrode was always calibrated in standard pH 7 and 1 solutions before each exam.

The distal electrode was placed 3 to 4cm above the inferior sphincter, according to Strobel's formula12 (equal to about 87% of the distance between the nostril and the inferior sphincter of the stomach), adapted and modified by Koda^13^ for children over 1 meter high.

The electrode position was always confirmed by a plain chest radiograph. The distal electrode should remain within the upper and lower borders of the 3rd vertebra above the diaphragm during inspiration and expiration.

The space between the proximal and distal electrode was set according to the age of the patient:^14^,^15^ 5cm separation (for patients aged under 1 year), 7.5cm separation (for patients aged between 1 and 8 years) 10cm separation (for patients aged 9 years or above).

The reference sensor was fixed to the thorax of the patient with an appropriate adhesive tape after applying conducting gel.

Collected data were stored in the memory of an Alacer Biomédica model Al-1 electronic recorder. A pH value was measured and recorded in the device memory every 4 seconds; stored data were later transferred to a computer. Maximum monitoring time was 24 hours. Data were analyzed with the dedicated “AL-1 Sistema de pH-metria versao 1.15” software (Alacer Biomedica).

We took age into account to assess prolonged pH monitoring in children.

Data were analyzed and compared according to Vandenplas's criteria^16^,^17^ for children up to age 2 years, and according to DeMeester scores^18^, ^19^, ^20^ for children over age 2 years.

DeMeester's Score Table was created by the author (DeMeester, TR) to define a scoring system for physiological and pathological reflux, and includes the following factors on Table 1:

**Table 1:** De Meester's Score Table.

Duration (HH:MM) 23:59
Number of episodes 50 3.4
Number of episodes >5 3 2.8
Longest episode (min) 12.0 1.7
Total time pH <4 (%) 3.2 2.2
Time pH <4 orthostatism (%) 3.6 1.6
Time pH <4 supine (5) 1.2 1.6
De Meester Score 13.3

There are 6 parameters:
(1) number of refluxes, (2) number of prolonged refluxes, (3) longest reflux, (4) total acid exposure time, (5) total acid exposure time while standing, (6) total acid exposure time while lying down.

All individuals with scores below 14.72 (95th percentile) were considered as having physiological reflux. The most commonly accepted definition of reflux is when there is a score below 4.0 lasting for over 15 seconds. The significance of DeMeester's score is that is separates patients with physiological reflux from those with pathological reflux.

Pathological reflux was considered as at least 1 reflux episode with a pH below 5 in the proximal sensor. Distal sensor reflux was considered “physiological distal reflux” if scores were within normal limits, and “pathological distal reflux” for scores above the normal range. In this case, patients were further classified according to orthostatism (predominance of reflux while standing), supine (predominance of reflux while lying down), and combined (altered reflux parameters in both positions).

## RESULTS

Of 10 patients, 5 had physiological reflux according to their age, of which 4 had positive proximal sensor reflux (that is, reflux that reached the proximal sensor). The other 5 patients that had pathological reflux for their age also showed positive proximal sensor reflux. Only one patient had physiological reflux according to age and a negative proximal sensor reflux (Table 2).

**Table 2:** Results of esophageal pH monitoring in patients with recurrent laryngeal papillomatosis.

											CONCLUSION	
	age	Distal GER	Prolonged GER	% total	%or-thostatism	%supine	distance from IES	DeMeester (normal < 14.72)	Proximal reflux	% proximal		
1.LLA*	4	36	7	10,7	15,0	6,8	12,5	39,4	20	4,6	Pathological distal reflux mixed type	Positive for proximal reflux
2.BA	12	59	7	10,5	16,4	0,0	15,0	36,1	34	3,2	Pathological distal reflux, orthostatic type	Positive for proximal reflux
3.ASS	7	57	4	6,6	9,7	2,2	12,5	21,8	31	3,8	Pathological distal reflux, orthostatic type	Positive for proximal reflux
4.JVCP	6	18	4	3,6	10,3	0,0	12,5	18,8	10	0,9	Pathological distal reflux, orthostatic type	Positive for proximal reflux
5.BFN	8	45	1	4,3	6,1	2,0	12,5	18,2	22	2,7	Pathological distal reflux, orthostatic type	Positive for proximal reflux
6.DPS	12	27	1	2,4	0,9	5,0	15,0	14,2	6	1,0	Physiological distal reflux for age	Positive for proximal reflux
7.EPL	7	15	1	2,4	0,3	4,0	12,5	13,1	6	1,1	Physiological distal reflux for age	Positive for proximal reflux
8.KAFS	3	21	0	2,2	3,3	1,0	12,5	10,4	8	0,7	Physiological distal reflux for age	Positive for proximal reflux
9.PLPS**	5	12	0	0,5	1,0	0,0	12,5	4,9	0	1,0	Physiological distal reflux for age	NEGATIVE for proximal reflux
10.JVMOS	3	7	0	0,3	0,6	0,1	12,5	3,3	2	0,1	Physiological distal reflux for age	Positive for proximal reflux

* tracheostomized patient

** patient under control of recurrences for over 2 years.

## DISCUSSION

Although there are few published papers on this theme, it is possible that gastroesophageal reflux is one of the determining factors in the manifestation, aggressiveness, and recurrence of RLP. We know that HPV may be found in its latent state in the apparently normal respiratory epithelium of RLP patients.^2^ Epithelial damage might ensue from reflux material causing direct irritation or damage, facilitating the appearance of papillomatous lesions, worsening the clinical picture and leading to recurrence. Thus, effective diagnosis and treatment of extraesophageal reflux would be important for all RLP patients where disease control is difficult.^11^

We decided to use 24-hour double probe (proximal and distal) ambulatory esophageal pH monitoring as this test provides reliable diagnostic confirmation if done adequately and interpreted correctly.^14^,^15^

Although 50% of our patients had physiological distal gastroesophageal reflux, and 90% had a positive proximal reflux, there are still many controversies about what should be considered pathological reflux, particularly in children. In our RLP patients, 90% had positive proximal reflux. Holland et al. demonstrated that 100% of 20 RLP patients had gastroesophageal reflux.21 In our series we noted that the only patient (number 9, table) that did not have pathological proximal reflux was also the patient with the longest recurrence-free period. Furthermore, the patient with the highest degree of proximal and combined reflux (number 1, table) was a tracheostomized patient where control of recurrence was difficult, suggesting that reflux may facilitate the recurrence of papillomas.

Holland et al., in 2002, divided 20 patients into two groups, one treated for reflux and the other left untreated. In the treated group there was a statistically significant decreased incidence of RLP surgery complications.^21^

Bradford studied 31 RLP patients in 2003 and found that 42% of patients had normal esophageal acid exposure and altered pharyngeal acid exposure.^22^ All of the patients presented evidence of at least one episode in which the pH was below 4 in the larynx, for which they were treated. In our study we noted pathological distal reflux in 50% of patients; but 90% had proximal pathological reflux. As Bradford, we also concluded that treatment for reflux shound be considered for RLP patients.^22^

Other authors have found a potential relation between the severity of RLP and gastroesophageal reflux.^8^,^11^ Furthermore, recent papers have shown that most of the surgical complications of RLP patients, such as scars, laryngeal membranes, and airway stenosis are late complications and tend to be more significant and frequent in patients with gastroesophageal reflux.^22^

Based on our study, we believe that the treatment of reflux may be a further treatment strategy to control this high morbidity disease. We intend to continue our study to identify possible changes in the clinical picture of these patients after treating gastroesophageal reflux.

## CONCLUSION

Our study allows us to conclude that there is a significant association between proximal gastroesophageal reflux and patients with RLP.
